# Long descending cervical propriospinal neurons differ from thoracic propriospinal neurons in response to low thoracic spinal injury

**DOI:** 10.1186/1471-2202-11-148

**Published:** 2010-11-23

**Authors:** Justin R Siebert, Frank A Middleton, Dennis J Stelzner

**Affiliations:** 1 Department of Cell and Developmental Biology, SUNY Upstate Medical University 750 East Adams Street Syracuse, New York 13210, USA; 2 Department of Neuroscience and Physiology, SUNY Upstate Medical University 750 East Adams Street Syracuse, New York 13210, USA

## Abstract

**Background:**

Propriospinal neurons, with axonal projections intrinsic to the spinal cord, have shown a greater regenerative response than supraspinal neurons after axotomy due to spinal cord injury (SCI). Our previous work focused on the response of axotomized short thoracic propriospinal (TPS) neurons following a low thoracic SCI (T9 spinal transection or moderate spinal contusion injury) in the rat. The present investigation analyzes the intrinsic response of cervical propriospinal neurons having long descending axons which project into the lumbosacral enlargement, long descending propriospinal tract (LDPT) axons. These neurons also were axotomized by T9 spinal injury in the same animals used in our previous study.

**Results:**

Utilizing laser microdissection (LMD), qRT-PCR, and immunohistochemistry, we studied LDPT neurons (located in the C5-C6 spinal segments) between 3-days, and 1-month following a low thoracic (T9) spinal cord injury. We examined the response of 89 genes related to growth factors, cell surface receptors, apoptosis, axonal regeneration, and neuroprotection/cell survival. We found a strong and significant down-regulation of ~25% of the genes analyzed early after injury (3-days post-injury) with a sustained down-regulation in most instances. In the few genes that were up-regulated (Actb, Atf3, Frs2, Hspb1, Nrap, Stat1) post-axotomy, the expression for all but one was down-regulated by 2-weeks post-injury. We also compared the uninjured TPS control neurons to the uninjured LDPT neurons used in this experiment for phenotypic differences between these two subpopulations of propriospinal neurons. We found significant differences in expression in 37 of the 84 genes examined between these two subpopulations of propriospinal neurons with LDPT neurons exhibiting a significantly higher base line expression for all but 3 of these genes compared to TPS neurons.

**Conclusions:**

Taken collectively these data indicate a broad overall down-regulation in the genes examined, including genes for neurotrophic/growth factor receptors as well as for several growth factors. There was a lack of a significant regenerative response, with the exception of an up-regulation of Atf3 and early up-regulation of Hspb1 (Hsp27), both involved in cell stress/neuroprotection as well as axonal regeneration. There was no indication of a cell death response over the first month post-injury. In addition, there appear to be significant phenotypic differences between uninjured TPS and LDPT neurons, which may partly account for the differences observed in their post-axotomy responses. The findings in this current study stand in stark contrast to the findings from our previous work on TPS neurons. This suggests that different approaches will be needed to enhance the capacity for each population of propriospinal neuron to survive and undergo successful axonal regeneration after SCI.

## Background

Most research on axonal regeneration after spinal cord injury has studied the regenerative ability of supraspinal neurons (SSNs), neurons located in the brain which have axonal projections into the spinal cord (i.e. corticospinal tract, respiratory bulbospinal, rubrospinal tract, vestibulospinal tract, and reticulospinal tract neurons). Particularly corticospinal tract axons have demonstrated a limited regenerative ability after spinal injury [1]. This is also the case for axons of other SSNs in the brainstem, particularly after axotomy at more caudal levels of the spinal cord (i.e. thoracic), although a regenerative response is sometimes found [2]. Regeneration is even limited when tested within environments known to be permissive to regeneration [3,4]. Some of the earliest studies showing that central nervous system axons have the capacity to regenerate in an appropriate environment were the seminal studies from Agauyo's laboratory showing propriospinal (PS) axons, damaged by nearby peripheral nerve graft insertion, were able to grow within peripheral nerve grafts [5,6]. Propriospinal axons had a more robust regenerative response in this instance than SSN axons, as well as in later studies using other permissive environments such as cellular bridges/grafts enriched in growth factors [7-9].

PS neurons, are a class of neuron intrinsic to the spinal cord, and have remained relatively understudied in regards to axonal regeneration. PS neurons can be divided into a variety of classes. Short thoracic PS (TPS) neurons arise from the thoracic levels of spinal cord and their axons project for a few segments in the rostral and caudal directions. TPS neurons primarily are involved in regulating axial musculature and postural mechanisms [10]. Long distance PS neurons, include long ascending propriospinal tract neurons found in the lumbosacral enlargement that project rostrally to the cervical enlargement, and long descending propriospinal tract (LDPT) neurons found in the cervical enlargement projecting mainly caudally to the lumbosacral enlargement. These different classes of long distance PS neurons send reciprocal projections between the limb segments and function in the regulation and fine-tuning of locomotion, limb coordination, and postural support, working in concert with SSNs [10]. In addition to the robust regenerative response that PS neurons exhibit following experimental axotomy [5-9,11,12], PS axons also appear to undergo a considerable amount of post-injury axonal plasticity. For instance, after partial surgical spinal injury, remaining intact propriospinal projections have been able to form functional neuronal bypass circuits caudal to the site of injury related to recovery of motor functions [12-14].

We recently studied the intrinsic response of TPS neurons using laser micro-dissection of pre-labelled TPS neurons at different periods (3-days, 1-week, 2-weeks, 1-month) after low thoracic spinal transection followed by gene microarray, qRT-PCR, and immunohistochemical analyses [15]. We identified a number of factors that may be related to differences in regenerative ability when comparing our findings with previous studies of SSNs. One difference was the large local inflammatory response seen 3-days post-axotomy in TPS neurons not described in most studies of SSNs after spinal injury [15]. In addition, many genes associated with axonal regeneration, and with a number of growth factor receptors (*i.e. *Gfra1, Ret, Lifr) were up-regulated acutely in axotomized TPS neurons [15]. We believe that the successful propriospinal regeneration found in previous studies [5-9] is the product both of this early regenerative response, as well as the ability to respond to neurotrophic/growth factors present in the implanted grafts. Besides sustaining regeneration, neurotrophic or growth factors present in the grafts may have protected locally axotomized TPS neurons from the strong apoptotic response seen in TPS neurons 3-days post-SCI in our previous study.

In the present investigation we analyzed pre-labelled LDPT neurons collected from the C5-C6 cervical spinal segments from the same animals used in our previous TPS study [15] using a similar genetic and immunohistochemical approach. We hypothesized that a similar robust regenerative response would be found in the LDPT neurons, but possibly at a more delayed period post-injury. However upon analysis, we were surprised to find a much smaller response to axotomy of LDPT neurons including the lack of a cell death or an obvious regenerative response, and down-regulation in many of the genes assessed by our qRT-PCR analysis. Instead of mounting the robust early response exhibited by TPS neurons, LDPT neurons appear to enter a state of relative dormancy or quiescence. These differences observed in the post-injury response led us to compare PCR array data from uninjured cervical controls in the present analysis, with PCR array data from uninjured thoracic controls available from our previous study [15]. This comparison revealed unexpected phenotypic differences between TPS neurons and LDPT neurons that may be one of several factors contributing to the differences in the post-axotomy response between these two populations of PS neurons.

## Methods

All procedures utilizing animals were approved by the SUNY Upstate Medical University Committee for the Humane Use of Animals, under the direction and guidelines of the institutional Department of Laboratory Animal Research and the Association for Assessment and Accreditation of Laboratory Animal Care.

Female hooded Long-Evans rats (N = 36, Simonsen; Santa Clara; CA) approximately 77 days (± 10 days) old were used in this study. Animals were assigned to various labelling, injury, and survival time-points (see Table 1 for group assignments).

**Table 1:** Animal and Experimental Group Assignments

Animal Group	N	Label	Injury	Type	Survival Time Post-injury	Total Survival Post-Labelling	Group Comparisons
1	4	FG	No	---	---	7 days	1 to 4
2	4	FG	No	---	---	2 weeks	1 to 5
3	4	FG	No	---	---	1 month	2 to 6
4	4	FG	Yes	Txn	3 days	10 days	3 to 7
5	4	FG	Yes	Txn	1 week	2 weeks	8 to 9
6	4	FG	Yes	Txn	2 weeks	3 weeks	
7	4	FG	Yes	Txn	1 month	5 weeks	
8	4	DTMR	No	---	---	2 weeks	
9	4	DTMR	Yes	Con	1 week	2 weeks	

**Animal Group Assignments**. Female Long-Evans rat were divided among 9 groups. Using the indicated tracer, Fluorogold (FG) or Dextran Tetramethyl Rhodamine (DTMR), TPS neurons were prelabelled by a series of bilateral injections into the upper lumbrosacral enlargement. Allowing 1-week for tracer transport, animals in groups 4-8 were subjected to either a T9 complete spinal transection (Txn) or moderate spinal contusion injury (25 mm weight drop, NYU Impactor; Con). Following spinal cord injury, animals were allowed to recover for the indicated period of time. The final column of this table indicates the time point comparisons that were made for both the gene and PCR validation studies conducted during this study

### Animal Surgeries

#### Retrograde Labelling of Cervical Long Descending Propriospinal Tract (LDPT) Neurons

Retrograde labelling of LDPT neurons was performed as previously described [15]. In brief, animals were anesthetized by an intraperitoneal injection of a ketamine/xylazine cocktail (0.07 cc/100 g). A laminectomy was made at vertebral level T-13 exposing the upper lumbosacral enlargement. Following exposure, six 0.30 μl Fluorogold (FG; Biotinum Inc; Hayward; CA; 3% w/v in dH2O) or Dextran Tetramethyl Rhodamine 3,000 M.W. (DTMR; Molecular Probes; Eugene; OR; 1% w/v in 1× PBS) injections were made bilaterally centered within the intermediate gray matter (laminae V - VIII) at the rostral, middle, and caudal aspects of the laminectomy site.

#### Spinal Transection

Spinal transection surgeries were performed one-week post-retrograde labelling, as described in [15]. In brief, following anaesthesia a laminectomy was performed at the T9 vertebral level. A pair of iridectomy scissors (Fine Scientific Tools; Foster City; CA) was used to completely transect the spinal cord. Following transection, a probe was scraped along the inner wall of the vertebral canal through the lesion site to further ensure a complete lesion.

#### Spinal Contusion

Using the same procedures that are described in detail in [15], some animals were subjected to a spinal contusion injury. In brief, animals were anesthetized, and a laminectomy was performed at the T9 vertebral level. Then a moderate spinal contusion injury was inflicted by dropping a 10 g rod from a height of 25 mm onto the exposed cord, using the NYU Impactor and MASCIS protocol [16,17].

#### Post Operative Care

Following all surgical procedures, the incisions were closed in anatomical layers, using 3.0 silk to close the musculature, and 3.0 Nylon to close the skin; external sutures were removed after the first post-operative week. All spinal injured animals had their bladders expressed twice daily until the micturition reflex returned, and injections of Cefazolin (Sandoz Inc; Princeton, NJ; 0.03 cc, s.q.) were given *b.i.d. *for the first week following spinal injury to prevent urinary tract infections, or when infections occurred. For the first 48-hours post-operatively Buprenorphine hydrochloride (Buprenex injectable; Ben Venue Laboratories Inc; Bedford, OH; 0.03 cc s.q.) was given *b.i.d. *for pain management. All post-operative animals had *ad libitum *access to both food and water.

### Histology and Immunohistochemistry

#### DTMR Tissue

Animals were euthanized by an injection of sodium pentobarbital (Fatal Plus, 0.5 cc), and transcardially perfused with 500 ml 0.1 M PBS (pH 7.4) followed by 500 ml 4% paraformaldehyde (pH 7.4). Spinal cords were post-fixed in 4% paraformaldehyde for at least 24-hours followed by cryoprotection in 20% sucrose for a minimum of 24-hours. Tissue samples were embedded in O.C.T compound (Tissue Tek^® ^embedding medium) and frozen on dry ice. Tissue was sectioned on a cryostat in the transverse plane at a thickness of 20 μm. Slides were maintained at -20°C until further processing.

#### Immunohistochemistry

DTMR and FG retrogradely labelled LDPT neurons were probed immunohistochemically for the expression of ATF-3 (ATF-3, #H-90, Santa Cruz Biotechnology; Santa-Cruz, CA).) 1:250. Slides for ATF-3 labelling were subjected to an antigen retrieval step (15-min at 95°C in Citric acid buffer; pH = 6.0) prior to incubation in the primary antibody over night at 4°C. Secondary antibody detection of ATF-3 labelling utilized a goat-anti-rabbit-AlexaFluor488 (1:500; Invitrogen; Carlsbad; CA). All sides were coverslipped with Vectashield (Vector Labs Inc; Burlingame; CA). DTMR retrogradely labelled LDPT neurons were also probed for signs of cell death using a TUNEL assay kit (R&D Systems; Minneapolis, MN) following the provided protocols.

#### Fluorescent Microscopy

All immunohistochemistry was visualized on a Zeiss Axio Imager A.1 microscope (Carl Zeiss; Germany). DTMR labelling was viewed under a CY3 filter, FG labelling under a UV filter, and the immunofluoresence was visualized under a FITC filter. All images were captured using a SPOT RT slider camera, model 2.3.1 (Diagnostic Instruments; Sterling Heights; MI). All digitized images where processed in the Spot™ Advanced software (v. 3.3.4 for Macintosh, Diagnostic Instruments Inc.), and adjusted for both image brightness and contrast. No other manipulations were made to these images.

### Laser Microdissection and Expression Analysis of LDPT Neurons

#### FG Tissue

Following appropriate post-operative recovery time, animals were euthanized by an i.p. injection of sodium pentobarbital (Fatal Plus, 0.5 cc) and then decapitated. The lower half of the cervical enlargement, C5-C7, was rapidly dissected, embedded in O.C.T compound (Tissue Tek^®^), and frozen on dry ice. Tissue samples were stored at -80°C until processing. Tissue sections were cut on a cryostat transversely at 16 μm and mounted on poly-ethylennaphtalae (PEN) foil slides (Leica; Wetzar; Germany). Slides were maintained at -20°C during the sectioning process and stored at -80°C overnight prior to laser microdissection (LMD).

#### LMD

Dissection of individual retrogradely labelled LDPT neurons was conducted in the manner described in [15]. In brief, sections on PEN foil slides were removed from storage and maintained on dry ice until used for LMD. Slides were positioned on the stage of a Leica AS LMD microscope (Leica Microsystems; Bannockburn; IL) and neurons located within the intermediate gray matter (Lamina V-VIII and X) were dissected over a period of 10-minutes and collected into nuclease-free PCR tubes containing 30 μl RLT Lysis Buffer (Qiagen, Valencia; CA) with 1% β-marcaptoethanol (Sigma Aldrich; St. Louis; MO). A minimum of 200 FG labelled neurons were collected from each animal.

#### RNA Purification & Amplification

Neurons collected by LMD were sent to our institution's microarray core facility for purification and amplification. Total RNA was purified using the RNeasy Mini kit (Qiagen; Valencia, CA). The RNA concentration and quality was determined by loading 1 μl of each sample onto an RNA 6000 Pico Chip (Agilent Technologies).

#### qRT-PCR

The reverse transcriptase (RT) reaction to convert the RNA into first strand cDNA for PCR was carried out using the RT^2 ^First Strand Kit (SA Biosciences; Frederick; MD) following the manufacturer's directions and the supplied reagents for each RNA sample. PCR was performed using the RT^2 ^SYBR Green qPCR Master Mix and RT^2 ^Profiler™ PCR Array for Rat Neurotrophins and Receptors (SA Biosciences; Frederick; MD), according to manufacturer's instructions. Additional custom PCR primers were designed for 28 additional specific genes of interest (GOI), using the Primer3 software [18] (http://frodo.wi.mit.edu/primer3; See Table 2) and ordered from Eurofins MWG Operon. RNA for these custom-designed PCR reactions was converted into cDNA and pre-amplified for PCR using the Ovation ™ RNA Amplification System (NuGen; San Carlos; CA). These PCR reactions were performed using a SybrGreen I Master Mix (Roche). Both the SA Biosciences PCR arrays and SybrGreen I PCR reactions were run in duplicate for each cDNA template on the LightCycler 480 (Roche).

**Table 2:** PCR Primers

Gene Symbol	Accession #	Left Primer	Right Primer	Product Size
Abcb5	XM_234725	CTGATAGAGCATGGCTTTGAATG	GGTTGTTTTATGGCAGAGCAGA	77
Akt3	NM_031575	TGGAGAGGAAGAGATGGATGC	TCCACTTGCCTTCTCTCGAAC	130
Arg1	NM_017134	TGGAACAATCAGTGTGGTGCT	ATCCACCCAAATGACGCATAG	103
Atf3	NM_012912	AATTGCTGCTGCCAAGTGTC	CAGTTCGGCATTCACACTCTC	94
Atg9a	NM_001014218	CCTTTGCGCAGATGGACGTT	ACAACTCGGTCTTCCCATCCT	107
Atg9b	XM_575327	CATTCTACCCTCAGCCCAGT	GAAGAGATTGCAGACCGAGC	121
Atrn	NM_031351	CGTGTGGTCATGTTGGTCA	GCACTAGAGCACCTTGAGTTTG	121
Casp2	NM_022522	GTGGAATGCATCCTGACCAT	TAACAGTTCGCTCAGCAGCA	83
Crem	S67786	CTGGCCAAATTTCTGTCCCTA	ACACCTTGTGGCAAAGCAGTA	133
Dbh	NM_013158	GGGATGTCCTCATCACTTCG	GTGGCCATTGTCCTGTTTTC	55
Flna	XM_238167	ACCAACCAACAGTGCAGACC	TGTTTGCTGGCTACCCTGAG	80
Fuk	XM_226508	AGAGCTCAGGCTCTAGGATGC	ACACCACCCATTGCCTATGA	112
Grm4	NM_022666	CCAATGTGCCATCCTCAGA	CCTACACCAACCATGCCATC	119
Itga6	XM_215984	GCCCTATGAACTTGGTGGAGA	AATACCCTTCCCTGAGTCCACA	100
Itgb1	NM_017022	CCACAACAGCTGCTTCTAAAGTTG	AATAGGGTAGTCTTCAGCCCTCTTG	84
Maob	NM_013198	TGGTGGATCTGGTCAAGTGAG	TCTCCTGTCTGGTCAATGTGG	96
Notch3	NM_008716	TGCAGGGTAGGCTTGGTATG	TGTGTCCAGACTGGGGCTTA	79
Nrap	NM_001107443	GGCGGCTCTTTTGGCTATC	GGGCAGCAGAGAGGGAAAG	72
Oaz1	NM_139081	AGTCAGCGGGATCACAGTCTT	AGGACCCTGGTCTTGTCGTTA	98
Pdgfra	XM_214030	CGAAGGCAGGCACATTTATATC	TATGATGGCACAATCGTCCTCT	109
Pycard	NM_172322	GCATACAGGAGCTGGCTGA	CAATGAGTGCTTGCCTGTGT	135
Rhoq	NM_053522	CTATGCTAACGACGCCTTCC	GATAAAGGCCTCAGACGATCA	147
Sat	NM_001007667	TGACCCATGGATTGGCAAGT	GCAGCGACACTTCATAGCAA	124
Slc27a3	XM_215605	CAGCTCCCCAATGTACTGGA	TAGGGGCCACTGTGGTACTG	101
Slc6a11	NM_024372	CCCGTCTTCTTCCTGGAAAC	GGACAGACTCTCCTCCAGCA	77
Tgfbr1	NM_012775	TTCATTTCAGAGGGCACCAC	CAATGGTCCTTGCAATTGTTC	109
Xiap	NM_022231	GAATATGACGCACGGATCGTT	CCTCCTCCACAGTGAAAGCAC	128
Zfp53	XM_344861	GCAAACAAGCTGCAGAGTCA	TGAGGCGGTACTGAAGCATT	132

**PCR Primer Table**. Primer sequences for all of the custom designed PCR primers used in this study. Primers were designed using the Primer3 software and synthesized by Eurofins MWG Operon (see Methods)

#### qRT-PCR Data Analysis

**Figure 1:**
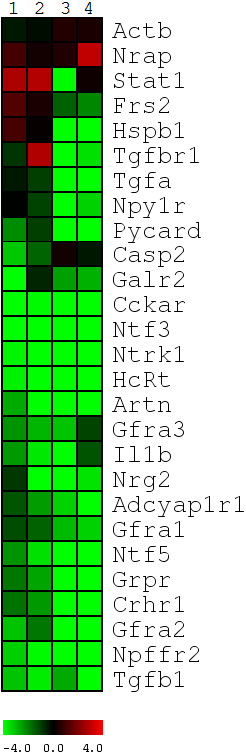
**Genes exhibiting a significant change in expression post-axotomy**. The expression profiles illustrate the normalized -ΔΔCp expression values compared to controls, with low expression values being represented by green and high expression in red. Significant changes in expression were determined using a 2-factor ANOVA, and then cluster analysis to reveal patterns in the expression profiles 3-days, 1-week, 2-weeks, and 4-weeks post-low thoracic spinal transection, numbered 1-4, respectively. As illustrated in this heat map, 19/27 genes exhibited an immediate down-regulation following axotomy. 18/19 genes remained down-regulated following axotomy except Casp2 which exhibited a slow return to baseline level at 1-month post-injury (column 4). Only two genes, Actb and Nrap, both of which are actin-cytoskeletal related, exhibited an up-regulation following axotomy which was maintained throughout the interval examined. The remaining 6 genes, Stat1, Frs2, Hspb1, Tgfa, Npy1r, and Tgfb1, either exhibited no response, or an up-regulation following axotomy, followed by a significant down-regulation by 2-weeks post-injury (column 3).

The average number of cycles required for a gene to reach the crossing point (Cp) above background, in both the uninjured controls and spinally transected animals, was calculated and then compared to the mean Cp of one or more reference genes included on the PCR array (Rlpl1, Ldha). In the cases where we custom designed PCR primers, the average Cp values were compared to the Cp value of the reference gene glyceraldehyde 3-phosphate dehydrogenase (Gadph), to give us the ΔCp value. The ΔCp value for each gene of the spinally transected group was then subtracted from the mean ΔCp value of the control group to yield the ΔΔCp, from which the directional and fold changes in our GOI were determined. All non-reference genes that generated Cp values less than 40 cycles in at least two samples within a given lesion or control group (n = 89 out of 113) were subjected to a 2 way ANOVA (Treatment × Time) to detect those with a significant main effect of Treatment, or a significant Treatment × Time interaction. To correct for multiple testing, the significance threshold for these analyses was adjusted using the Benjamini-Hochberg False Discover Rate (FDR) set to 0.10. After FDR correction, the expression data for all genes with a significant main effect of Treatment (n = 27; Table 3) were displayed in a heat map and subjected to hierarchical clustering (Figure 1).

**Table 3:** Genes Exhibiting Significant Change in Expression Post-Axotomy

Gene	Treat F	Treat P	Treat FDR	Time F	Time P	Time FDR	Intx F	Intx P	Intx FDR	Gene Name
Tgfb1	39.26	0.0008	0.012	0.37	0.7775	0.975	0.29	0.8294	0.959	Transforming growth factor beta-1
Gfra2	58.9	0.0006	0.013	18.01	0.0041	0.182	5.2	0.0537	0.478	GDNF family receptor alpha-2
Artn	53.09	0.0008	0.014	2.46	0.1782	0.721	3.22	0.1205	0.564	Artemin
Tgfbr1	23.1	0.0005	0.015	11.2	0.0011	0.098	17.34	0.0002	0.018	TGF-beta receptor type-1
Crhr1	39.2	0.0015	0.017	0.94	0.489	0.907	0.92	0.4934	0.878	Corticotropin-releasing factor receptor 1
Npffr2	40.59	0.0014	0.018	3.18	0.1223	0.544	0.47	0.7164	0.996	Neuropeptide FF receptor 2 (G-protein coupled receptor 74)
Ntf5	166.32	0.0002	0.018	17.61	0.0091	0.162	14.58	0.0128	0.163	Neurotrophin 4/5
Nrap	17.68	0.0023	0.019	1.13	0.3895	0.889	3.53	0.0618	0.458	Nebulin-related-anchoring protein
Adcyap1r1	32.85	0.0023	0.02	3.25	0.1185	0.555	0.84	0.5267	0.868	Pituitary adenylate cyclase-activating polypeptide type I receptor
HcRt	63.71	0.0005	0.022	8.2	0.0224	0.285	1.89	0.2498	0.717	Orexin (Hypocretin)
Npy1r	29.78	0.0055	0.038	17.98	0.0087	0.194	19.13	0.0078	0.116	Neuropeptide Y receptor type 1
Gfra3	22.14	0.0053	0.039	0.85	0.5251	0.935	9.31	0.0173	0.192	GDNF family receptor alpha-3
Frs2	19.94	0.0066	0.042	17.85	0.0042	0.125	21.47	0.0028	0.083	Fibroblast growth factor receptor substrate 2
Grpr	17.86	0.0083	0.043	0.32	0.8116	0.976	0.71	0.5889	0.92	Gastrin-releasing peptide receptor
Gfra1	16.98	0.0092	0.045	0.34	0.8009	0.976	0.39	0.767	0.922	GDNF family receptor alpha-1
Ntf3	24.74	0.0076	0.045	1.72	0.301	0.893	4.94	0.0785	0.466	Neurotrophin-3
Ntrk1	23.83	0.0081	0.045	8.06	0.0359	0.32	0.45	0.7318	0.972	High affinity nerve growth factor receptor (Trk-A)
Cckar	15.25	0.0113	0.053	0.96	0.4787	0.906	0.46	0.7241	0.991	Cholecystokinin type A receptor
Pycard	9.5	0.0151	0.058	0.52	0.6783	1	1.69	0.2448	0.726	PYD and CARD domain-containing protein
Casp2	9.48	0.0132	0.059	1.38	0.3117	0.895	1.44	0.2955	0.797	Caspase-2
Hspb1	13.15	0.0151	0.061	4.16	0.0794	0.471	15.58	0.0057	0.127	Heat-shock protein 27
Tgfa	11.48	0.0147	0.062	0.31	0.8191	0.959	1.99	0.2174	0.774	Transforming growth factor alpha
Stat4	9.45	0.0218	0.081	0.28	0.8397	0.934	0.08	0.9705	0.993	Signal transducer and activator of transcription 4
Galr2	7.47	0.0257	0.088	4.63	0.037	0.299	1.08	0.4106	0.937	Galanin receptor type 2
Nrg2	6.98	0.0247	0.088	1.19	0.364	0.926	0.61	0.6221	0.955	Pro-neuregulin-2, membrane-bound isoform
Il1b	11.62	0.0271	0.089	2.78	0.1742	0.738	0.91	0.5119	0.876	Interleukin-1 beta

**P values for genes exhibiting a significant or nominally significant change in expression post-axotomy**. Significant changes in gene expression post-transection were detected using a 2 way ANOVA. As illustrated above, genes were analyzed for a significant effect of treatment (Treat), Time (Time), and Interaction of Treatment and Time (Intx). P-values were then corrected for multiple testing applying the Benjamini-Hochberg FDR algorithm, and are arraigned in order of increasing FDR values based on treatment

## Results

### Overall Response of LDPT Neurons to a T9 Level Spinal Cord Injury

A 2-way ANOVA (Treatment × Time) was used to determine which of the 89 genes that could be analyzed from our original data set (see qRT-PCR, Methods) exhibited a significant change in expression due to the main effect of treatment (See Methods). Following correction for multiple testing (see qRT-PCR Methods) only 18 were found to be significant to the *P ≤ 0.05 *level, while an additional 9 genes reached a level of nominal significance (See Table 3). When the -ΔΔCp values were determined to indicate direction of change, the results were unexpected. As illustrated in Figure 1, 13 of the 18 genes that demonstrated a significant change in expression and 6 of the 9 genes that had a nominally significant change exhibited an immediate down-regulation post-axotomy. Of these 19 genes showing an immediate down-regulation, 2 are related to apoptosis (Casp2, Pycard); 4 are neuropeptides (Cckar, HcRt, Grpr, Npffr2), regulate energy metabolism (HcRt), pain modulation (Grpr, Npffr2), and endorphin/dopamine release (Cckar); 7 are neurotrophic factor or other surface receptors (Adycap1r1, Crhr1, Galr2, Gfrα1, Gfrα2, Gfrα3, and Ntrk1 (TrkA)); and 6 are neurotrophic agents (Artn, Il1b, Ntf3 (NT-3), Ntf5 (NT-4/5), Nrg2, and Tgfb1). Interestingly, after the initial down-regulation of these genes, only Casp2 showed a gradual increase in expression back towards control levels by 1-month post-axotomy (column 4 in Figure 1).

**Figure 2:**
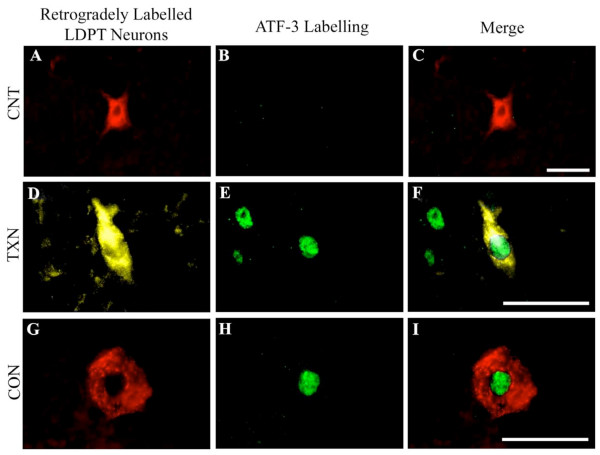
**Post-injury expression of ATF-3**. One gene of interest that was examined using PCR was ATF-3. However due to the lack of PCR reaction product in the control animals and presence of PCR reaction product in the experimental animals, calculation of -ΔΔCp values and statistics was problematic. Therefore we probed for expression of ATF-3 protein immunohistochemically. ***A, C: ***In uninjured control animals a DTMR labelled LDPT neuron is clearly visible (red) and there is a lack of any ATF-3 labelling (**B**; green). ***D, E, F ***However, when an animal that received a complete T-9 spinal transection was examined 4-days post-injury, the FG labelled LDPT neuron (**D; **in yellow) colocalizes with the nuclear ATF-3 labelling shown in green (**E, F**). ***G, H, I ***Moreover, when animals that received a T-9 spinal contusion injury are examined 1-week post-injury, the DTMR labelled LDPT neurons (**G; **in red), again, colocalize with the ATF-3 labelling (**H; **in green). These findings complement the PCR findings indicating an up-regulation of ATF-3 post-SCI. CNT = Uninjured Control, TXN = Transection, CON = Contusion. Scale Bars = 50 μm

While a majority of the genes significantly affected by axotomy were down regulated, 4 genes exhibited significant up-regulation following axotomy, a transcription factor (Stat1), a growth factor receptor component (Frs2), an actin-cytoskeletal related protein (Nrap), and a cell stress and axonal regeneration associated gene (Hspb1) (Figure 1). The expression profiles for Stat1, Frs2, and Hspb1, showed an immediate up-regulation at 3-days post-injury (column 1) and 1-week post-injury (column 2), but by 2-weeks post-injury expression of these 3 genes was down-regulated (column 3). Nrap was the only gene that remained up-regulated, with maximal expression appearing 1-month post-injury (column 4). Atf3 also demonstrated a significant up-regulation post-axotomy but is not indicated on the heat map (Figure 1). There was a lack of any detectable PCR product when probing for this gene in the uninjured controls (resulting in no Cp values), which made calculating -ΔΔCp values and the accompanying statistics problematic. This lack of Atf3 PCR product in the controls and presence in spinally injured animals was interpreted to indicate that Atf3 mRNA was not expressed at a measurable level in uninjured animals, but was significantly up-regulated following injury. The up-regulation of ATF-3 protein was demonstrated in both the spinally transected and contused animals. As illustrated in Figure 2A-C, DTMR retrogradely labelled LDPT neurons are clearly visible (Figure 2a) and there is a lack of any ATF-3 immunolabelling (Figure 2B,C) in uninjured control animals. However, when animals that received a low-thoracic spinal transection (Figures 2D-F), or spinal contusion (Figures 2G-I) were examined for ATF-3 activation (examined at 4-days and 1-week post-injury) there was pronounced ATF-3 immunolabelling (Figures 2E and 2H). As illustrated, retrogradely labelled LDPT neurons (Figures 2D, and 2G) colocalize with the clear nuclear ATF-3 immunolabelling (Figures 2E, and 2H) when the images are overlaid (Figures 2F and 2I). This immunofluorescent staining experiment confirmed our PCR findings, in which ATF-3 expression is only found in the spinally injured animals.

The remaining 4 genes (Npy1r, Tgfa, Actb, Tgfbr1) were relatively unaffected at early times post-injury, but exhibited a significant change in expression over time. As illustrated in Figure 1, the mRNAs for neuropeptide receptor (Npy1r) and growth factor (Tgfa) are slightly down-regulated at 1-week post-injury, but are then strongly down-regulated at later survival times. The mRNA for β-actin, an actin cytoskeletal element, is up-regulated at the later time points post-injury (columns 3 and 4; Table 3 shows this up-regulation most clearly). This up-regulation of Actb has been noted in a number of investigations post-axotomy [19-21]. The most unique expression pattern of the 4 genes was for the surface receptor Tgfbr1, which was significantly down-regulated post-injury, and then had a complicated pattern of expression at other survival times.

### LDPT Neurons Appear to Lack a Cell Death Response Following Low Thoracic Spinal Cord Injury

The expression of a number of genes involved in a pro- or anti-apoptotic response was examined using a series of PCR primers either included on the array plate, or custom designed. These primers included Bax, Casp2, Myc, Ngfrap1, Pycard, and Tp53, all of which are pro-apoptotic [22-30], while Akt3, Bcl2, Il10, and Xiap are considered to be anti-apoptotic [31-36]. Only two of these genes were affected by axotomy/thoracic spinal injury. As shown in Table 3, Pycard (P = 0.058) and Casp2 (P = 0.059) showed a nominally significant change in expression post-axotomy. Moreover, as shown in Figure 1, both Pycard and Casp2 were down-regulated. Pycard remained down-regulated throughout the period examined, whereas the levels of Casp2 showed a gradual increase in expression, approaching baseline levels (indicated by black) at later survival times. In addition to the down-regulation of these two pro-apoptotic elements, the lack of any change in expression of the other pro-apoptotic or anti-apoptotic genes strongly suggests that LDPT neurons are not mounting a pro- or anti-apoptotic response to a T9 level axotomy over the time course examined. Additionally, two genes regulating the formation of autophagic vesicles, signs of homeostatic stress or autophagocytosis, Atg9a and Atg9b [37,38], also failed to show a significant change in expression over the time course examined post-axotomy.

**Figure 3:**
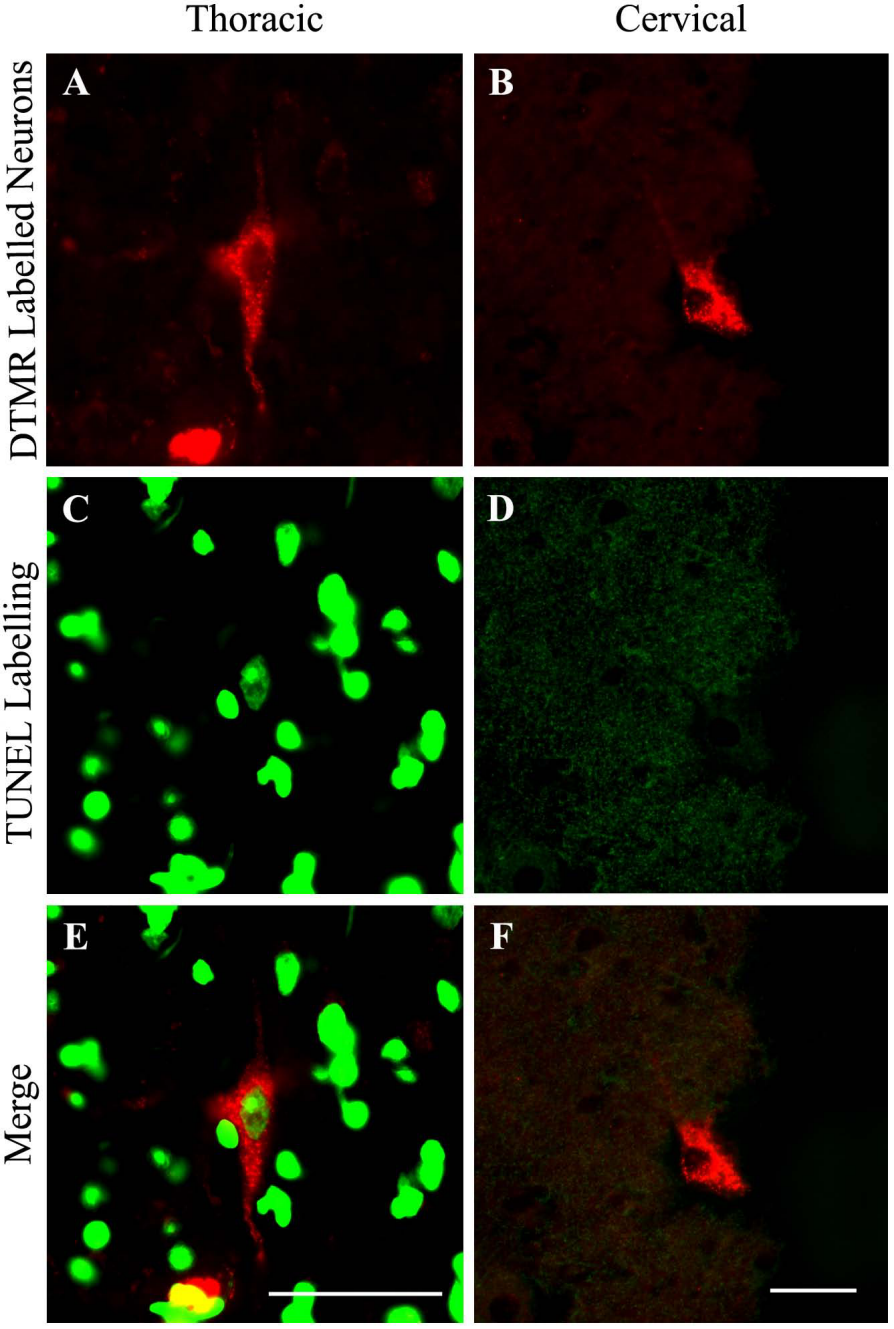
**TUNEL Staining**. To further explore the difference in the cell death response that was observed in the LDPT neurons compared to what was found in our previous TPS study, we utilized a TUNEL assay kit to examine for signs of cell death 1-week following T9 level contusion. ***A, C, E*, **DTMR retrogradely labelled TPS neurons probed with the TUNEL assay kit show colocalization of a TUNEL labelled nucleus within a retrogradely labelled TPS neuron, additionally, multiple TUNEL labelled nuclei are present in the merged image, possibly glial cells or interneurons, not retrogradely labelled by TMR. ***B, D, F*, **However, when cervical spinal cord is examined, while DTMR retrogradely labelled LDPT neurons are clearly visible, no signs of TUNEL labelling are observed in neurons, or in the surrounding tissue. Scale Bars = 50 μm

To further examine the evidence for an injury-induced apoptotic response, we also analyzed retrogradely labelled neurons immunohistochemically using the TUNEL assay. This was performed on tissue sections taken from C5-C6 spinal segments containing labelled LDPT neurons and the T6-T7 spinal segments containing labelled TPS neurons 1-week following a T9 moderate spinal contusion injury. As illustrated in Figure 3, there was co-localization of TUNEL staining within labelled TPS neurons (Figure 3A, C, E). This result supports our previous findings showing a strong early cell death response of TPS neurons following T9 level injury [15]. However sections from the cervical enlargement had little TUNEL labelling and little to no co-localization of the TUNEL labelling within retrogradely labelled LDPT neurons (Figure 3B, D, F). The TUNEL findings complement the PCR data, and further argue that LDPT neurons do not undergo a significant amount of post-axotomy retrograde cell death.

### Phenotypic Differences Exist Between Uninjured TPS and LDPT Neurons

The results of the present study indicate that LDPT neurons respond very differently compared to TPS neurons [15] after the same low thoracic spinal transection. Based on these observations, we were prompted to determine if there were any fundamental baseline differences between these two populations of PS neurons that could underlie their strikingly different transcriptional responses to the same injury. To begin to examine this issue, we compared the PCR array plate data from neurons collected from uninjured cervical controls and uninjured thoracic controls from our previous study [15]. Since the RNA samples were collected and purified in the same manner, and the RT reaction and PCR array plates were identical for both studies (see Methods), we were able to compare the previously generated Cp values from the thoracic control animals with the cervical control animal Cp values generated in the present study.

The analysis of the control PCR data was conducted in a manner similar to that already mentioned in the Methods section with some minor modifications. In brief, once all Cp values were determined, they were compared to the average Cp of four reference genes included on the PCR array (Hprt, Ldha, Rplp1, and Rpl13a) to give us the ΔCp value. Then for each gene the ΔCp value of the thoracic control group (TPS neurons, retrogradely labelled for 1-week, 2-weeks, or 1-month after injection of FG) was subtracted from the mean ΔCp value of the cervical control group (LDPT neurons, retrogradely labelled for the same time periods as TPS neurons) to yield the ΔΔCp. The ΔΔCp values were then used to indicate the direction and fold differences in gene expression between the uninjured LDPT and TPS neurons that served as controls in the present and previous study, respectively.

The PCR array data were analyzed using a 2 way ANOVA (Treatment × Time) to detect genes with a significant main effect of level. The significance threshold for these analyses then was adjusted using the Benjamini-Hochberg FDR set to 0.10. Genes with a significant main effect of level were subjected to a Welch's t-test post-hoc to determine at which specific time points (1-week, 2-weeks, or 1-month post-FG labelling) the difference in expression was significant. The unadjusted P-values, FDR corrected P-values and post-hoc test P-values were then compiled and are presented in Table 4.

**Table 4:** Phenotypic Differences Between Thoracic and Cervical Propriospinal Neurons

					Fold Chg	Individual Fold Chg			Post hoc P value		
Gene	Level F	Level P	FDR	ΔΔCp	Cerv-Thor	Time1	Time2	Time3	Time1	Time2	Time3
Hspb1	159.50	0.00002	0.001	-3.67	12.8	0.9	43.2	50.9	0.9273	***0.0426***	0.0833
Lifr	142.15	0.00002	0.001	-3.31	9.9	7.2	6.3	21.7	0.062	***0.0273***	***0.0029***
Ldha	104.55	0.00005	0.001	3.06	0.1	0.1	0.2	0.1	***2E-05***	***0.0003***	***0.0062***
Rplp1	82.46	0.00010	0.001	-3.55	11.7	10.2	8.9	17.7	***6E-06***	***0.0004***	***0.0001***
Nf1	53.07	0.00030	0.004	-2.29	4.9	1.4	4.0	20.6	0.7123	***0.0375***	0.0509
Ntf5	52.54	0.00040	0.004	-5.52	46.0	14.5	48.6	138.2	***0.0027***	***0.0139***	***0.0107***
Crhr1	34.54	0.00110	0.009	-5.69	51.5	7.8	88.0	198.9	0.0633	***0.0091***	***0.0073***
Gfra2	34.36	0.00110	0.008	-2.54	5.8	1.2	8.8	18.4	0.6241	0.1267	***0.0388***
Gfra1	26.31	0.00220	0.014	-3.29	9.8	3.8	12.1	20.5	0.3976	***0.0122***	***0.002***
HcRt	20.89	0.00380	0.022	-7.96	248.3	29.1	857.7	614.3	***0.0049***	***0.0159***	***0.0079***
Ntrk2	19.49	0.00450	0.024	-0.22	1.2	0.7	1.1	2.2	0.5642	0.9113	0.5394
Zfp110	19.21	0.00470	0.023	-2.74	6.7	2.9	8.5	12.3	0.2716	***0.0457***	0.1485
Tp53	17.61	0.00570	0.026	-2.49	5.6	5.1	5.5	6.3	0.1686	0.0759	0.1054
Tgfa	16.48	0.00670	0.028	-3.99	15.9	3.5	21.7	53.0	0.0804	***0.0008***	***0.0015***
Ntrk1	14.67	0.00870	0.034	-5.98	63.3	19.7	106.5	120.6	***0.0049***	***0.0006***	***0.0006***
Zfp91	14.44	0.00900	0.033	-1.70	3.2	1.4	3.0	8.0	0.5574	0.252	***0.0011***
Npffr2	13.86	0.00980	0.034	-4.59	24.1	9.3	19.3	78.0	***0.0283***	***0.0038***	***0.0007***
Artn	13.89	0.00980	0.032	-6.97	125.4	21.8	335.2	270.2	***0.0017***	***0.0482***	***0.0045***
Npy1r	13.61	0.01020	0.032	-5.44	43.3	4.9	89.2	184.5	0.1173	***0.0017***	***0.0002***
Fgfr1	12.28	0.01280	0.038	-1.33	2.5	1.6	3.4	3.0	0.3199	0.0554	0.1921
Stat1	11.92	0.01360	0.038	-0.54	1.5	0.2	8.0	1.9	0.2964	***0.0022***	0.7465
Fus	10.51	0.01760	0.047	-1.56	3.0	1.7	2.0	7.3	0.066	0.1028	***0.0051***
Cntfr	10.26	0.01850	0.047	-0.52	1.4	1.6	0.9	2.2	0.5502	0.8505	0.4669
Npy	10.16	0.01890	0.046	-1.45	2.7	0.9	2.9	8.2	0.8662	***0.0062***	***0.0459***
Frs2	9.82	0.02020	0.048	-1.22	2.3	0.8	3.4	4.8	0.8758	***0.0124***	0.3093
Adcyap1r1	9.22	0.02290	0.052	-4.42	21.5	9.0	27.6	39.9	***0.0268***	***0.0043***	***9E-05***
Hprt	9.22	0.02290	0.050	2.19	0.2	0.2	0.2	0.3	0.0719	0.0575	***0.002***
Ppyr1	7.79	0.03150	0.066	-6.86	116.2	31.2	69.5	723.5	0.1673	***0.0261***	***0.0019***
Tgfb1	7.65	0.03260	0.066	-3.84	14.3	13.9	7.9	26.9	***0.0056***	***0.0095***	***0.0071***
Mt3	6.94	0.03880	0.076	0.14	0.9	0.9	0.9	0.9	0.7328	0.4609	0.8946
Rpl13a	6.27	0.04630	0.088	-1.66	3.2	4.4	3.1	2.3	***0.0223***	***0.0435***	***0.0002***

**Phenotypic differences between Thoracic and Cervical Propriospinal neurons**. Significant differences in gene expression between LDPT and TPS neurons were detected using a 2 way ANOVA (shown in bold at Time 1, 1-week; Time 2, 2-weeks; and Time 3, 4-weeks post-FG injection). Genes were analyzed for a significant effect of level. P-values were then corrected for multiple testing, by applying the Benjamini-Hochberg FDR algorithm. A Welch's t-test, was used to determine at which specific time-point the difference in gene expression was significant. In the table above 2E-05 represents P values of 2 × 10^-5 ^and 2 E-06 represents a P value of 2 × 10^-6^. Significant post-hoc P values are shown in italics.

As illustrated in Table 4, 31 of the 84 genes on the PCR array plate demonstrated a significant difference in expression level between the cervical and thoracic samples. Eight of these genes encode neurotrophic/growth factor receptors (Lifr, Crhr1, Gfra2, Gfra1, Ntrk2, Ntrk1, Cntfr, and Adcyap1r1), and 7 encode agents that enhance neuronal survival or axonal regeneration (Hspb1, Ntf5, Tgfa, Zfp91, Artn, Tgfb1, and Mt3). Twelve of 16 of the other genes demonstrating a significant difference in expression regulate axonal branching (Nf1; [39]); are various neuropeptides/neuropeptide receptors (Hcrt, Npffr2, Npy1r, Npy, Ppyr1); regulate transcription (Fus, Stat1 and Zfp110); modulate cellular signalling (Frs2); regulate apoptosis (Tp53); or is involved with nervous system development (Fgfr1). One finding that was unexpected was the significant difference in expression level of the reference genes used on the array plate (Ldha, Rplp1, Hprt, and Rpl13a). Even though the average of the 4 reference genes was used to calculate the ΔCp values, they still exhibited a significant difference in expression between cervical and thoracic spinal cord. Since the other PCR Array plate control probe data (Rat Genomic DNA Contamination Control, Reverse Transcription Control, and Reverse Transcription Control) did not differ between plates used for the TPS and LDPT analyses, these data appear valid. As shown in Table 4, 28 of the 31 genes in the PCR array that were significantly different between propriospinal neurons collected from the thoracic or the cervical spinal cord were more highly expressed in cervical neurons. Only Ldha and Hprt, two of the reference genes, and metallothionein (Mt3) exhibited a higher expression level in thoracic neurons.

## Discussion

This study is the first to our knowledge to specifically study the post-axotomy response of LDPT neurons. Rather than focusing on the response during the first 24 hours post-axotomy, we began our analysis 3-days post-injury (p.i.), and continued at different periods up to 1-month p.i., over a longer time course than has been analyzed in most previous studies [11,40-44]. Just as was done in our TPS study, we used LMD to specifically collect individual FG retrogradely pre-labelled LDPT neurons at different times after axotomy. Therefore, the mRNA collected was from a relatively pure sample limited predominantly to LDPT neurons. Again as with our previous TPS study, one potential criticism is the use of Fluorogold (FG) to retrogradely pre-label our LDPT neurons. It has been suggested that FG may have cytotoxic effects on neurons over time [45,46]. However as reported in our previous study [15], no significant changes in gene expression were found comparing our control groups at the various post-FG labelling time points (1-week, 2-weeks, or 1-month). Moreover, there was no evidence (genetically or immunofluorescently) of a pro-apoptotic response in these control groups during the first month following SCI. These findings support the argument that FG labelling has no adverse effects on neurons, and is a suitable neuronal tracer for this type of study.

While this study did not utilize the gene microarray analysis used in our TPS study [15], we analyzed the post-axotomy response of LDPT neurons using qRT-PCR arrays and custom-designed primers for a number of categories of genes, including many of the same genes that were analyzed by qRT-PCR in the TPS study. Moreover, the spinal tissue examined was from the same animals, survival times, and treatment used in the previous study. Although the LDPT neuronal samples were analyzed at different times than the TPS samples, the tissue was prepared and treated together prior to the final analysis. An advantage of this type of investigation is that we can directly compare the present data with our previous findings of TPS neurons.

Most previous studies examining the genetic changes of SSNs post-SCI have utilized *in situ *hybridization. This method is able to resolve genetic changes at a cellular level but only is able to analyze a relatively small number of selected genes [47,48]. While the current study only utilized qRT-PCR to examine the changes occurring in LDPT neurons post-axotomy, the use of the PCR array plates in combination with custom-designed primers for 28 additional genes allowed us to evaluate the expression of 113 genes simultaneously (See Additional File 1), 89 of which were shown to be expressed in our LDPT samples.

### Differential Response of LDPT and TPS Neurons to T9 Axotomy

This study set out to investigate the intrinsic response of LDPT neurons to a T9 spinal cord transection. We hypothesized that LDPT neurons would respond in a manner similar to what we previously documented in TPS neurons [15], but in a delayed manner due to the greater distance between cell body and axotomy site. However, the results of this study clearly demonstrate that, instead of mounting the contiguous acute regenerative and apoptotic response seen in TPS neurons, LDPT neurons initiate an overall down-regulation of most of the significantly affected genes that were examined.

Of the 11 genes examined in the present study that are pro- or anti-apoptotic, the only two that were nominally significant (Pycard and Casp2) are pro-apoptotic but both were down-regulated. Anti-apoptotic genes including Akt3, Bcl2, Il10 and Xiap [31-36] were unaffected. Additionally, two genes involved in regulating the formation of autophagic vesicles, Atg9a and Atg9b, that can be involved in caspase-independent cell death [37,38], also failed to show a significant change in expression over the time course examined post-axotomy. This lack of a cell death response was also supported by little TUNEL immunostaining or co-localization of TUNEL immunoreactivity within retrogradely labelled LDPT neurons, at least 1-week post-SCI (Figure 3). TUNEL immunolabelling within retrogradely labelled TPS neurons at the 1-week time point in the present study also supports our previous findings of an early apoptotic response in TPS neurons post-SCI [15]. Moreover, the caspase 3 immuno-reactivity found in pre-labelled TPS neurons 1 week post-SCI in our previous study (15) is not observed 1 week post-SCI in pre-labelled LDPT neurons (unpublished observations).

An examination of the genes involved in cell stress/neuroprotection and axonal regeneration revealed a consistent up-regulation of Atf3, but only a transient up-regulation of Hspb1 (Hsp27) in LDPT neurons post-axotomy. Other genes encoding agents that enhance neuronal survival or axonal regeneration were unaffected (Zfp91, Mt3) or down-regulated (Ntf5, Tgfa, Artn, Tgfb1). ATF-3 is a transcription factor that is often elevated after axonal injury or inflammatory processes. ATF-3 is up-regulated in dorsal root ganglion (DRG) neurons after peripheral nerve injury, and is down-regulated once regeneration is complete [49]. HSP-27 is a molecular chaperone involved in a number of functions that promote neuronal survival, as well as promoting axonal regeneration [50-52]. Hsp27 is a downstream product of the dimerisation of ATF-3 and c-Jun, another transcription factor usually up-regulated after axotomy [53] that was not examined in the current study. c-Jun has been associated with apoptosis, neuronal survival, as well as regeneration [53]. The intrinsic growth state of DRG neurons is increased, and Hsp27 is more highly expressed in transgenic mice that over-express ATF-3 [49]. These data suggest that ATF-3 regulates Hsp27 and both are involved in promoting axonal growth. However, the divergence in the continued expression of ATF-3, and down-regulation of Hsp-27 at later time points in the current study differs from these previous findings [49,53], and suggests that other factors are involved. Further study is needed to determine the role of these genes in the LDPT post-injury response, as well as the level of expression of c-Jun at different times after axotomy. c-Jun has been implicated as a pivotal regulator of whether a neuron survives post-axotomy but does not undergo a strong intrinsic regenerative response, or a neuron initiates a strong regenerative response as well as a cell death response, similar to the result found for TPS neurons in our previous study [15,53].

**Figure 4:**
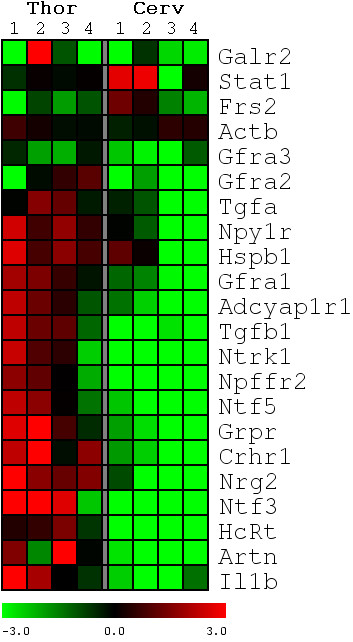
**Comparative post-injury response**. The expression profiles illustrate the normalized -ΔΔCp expression values of the post-injury response for both the TPS and LDPT neurons at the same survival times as in Figure 1 and numbered 1-4, with low expression values being represented by green and high expression in red. Significant changes in expression were determined using a 2-factor ANOVA, and then cluster analysis to reveal patterns in the expression profiles. As illustrated in this heat map, the differences in the post-injury response are striking. While many of the genes in LDPT neurons are down-regulated following a T9 level axotomy, the opposite is found in TPS neurons were there is a strong up-regulation following axotomy. The data illustrated in this heat map clearly demonstrate a differential response to a T9 level axotomy.

This stark contrast between the intrinsic LDPT and TPS response to axotomy is illustrated in the heat map shown in Figure 4. This heat map illustrates that while TPS neurons mount a robust post-axotomy response, most genes of LDPT neurons that were examined are down-regulated for the entire post-SCI period examined. These data suggest that LDPT neurons may enter a state of relative quiescence or dormancy. One gene in particular, hypocretin (orexin) neuropeptide precursor (HcRt), is known to regulate energy metabolism in neurons [54,55], among other functions, and, is significantly down-regulated in LDPT neurons but up-regulated in TPS neurons following axotomy. Several neurotrophic factors (Atrn, Ntf3, Ntf5) are down-regulated. There is the simultaneous down-regulation of genes for a number of neurotrophic factor receptors (Gfra3, Gfra2, Gfra1, Ntrk1, and Adcyap1r1). This down-regulation is likely to make LDPT neurons less responsive to these neurotrophic factors. A second possibility for the down-regulation of the surface receptors is that the decrease in mRNA expression is due to the stabilization of the receptor proteins which, in turn, could down-regulate new synthesis of these receptors. As Figure 4 shows, this down-regulation is completely different from the response of TPS neurons post-low thoracic SCI found in our previous study [15]. While these disparate effects of thoracic SCI/axotomy on LDPT and TPS neurons is quite clear, what remains unresolved is the reason for this differential response.

### Factors contributing to the differences in the intrinsic response of LDPT and TPS neurons to low thoracic axotomy

One likely possible explanation for the differential effects of low thoracic axotomy on the cellular response of LDPT and TPS neurons is the distance between the site of the axotomy and the cell body of the neuron [47,56,57]. The present study specifically analyzed neurons collected from the C5-C6 level spinal cord, 11 segments rostral to the lesion site, whereas TPS neurons were collected from the T6-T7 spinal level, two segments rostral (approximately 2 mm) to the lesion. With axotomy occurring at the T9 level, TPS neurons also are located near the lesion site with the associated inflammatory and immune responses that occur in this region, and many of the genes most highly up-regulated in the microarray analysis of TPS neurons post-injury were related to inflammation [15]. Previous work indicates one result of inflammation and invasion of vascular macrophages [48,56-63] is a neuroprotective response that also may be needed to stimulate a maximal regenerative response [62,63] fostering successful regeneration within PNS implants [64-66] and within the central nervous system, itself [48]. For this reason, it is difficult to separate the inflammatory effect of proximal injury, from the effects of axotomy close to the cell body being primarily responsible for the differential response comparing TPS and LDPT neurons in our analyses.

One study attempting to separate the inflammatory response from site of axotomy was conducted by Houssain-Ibraham and colleagues [48] in a study of corticospinal tract (CST) neurons. Lipopolysaccharide (LPS), an inflammatory reagent, was applied to the cerebral cortex near the cell bodies of spinally axotomized CST neurons. LPS resulted in increased expression of a number of regenerative associated genes (e.g., Gap-43, Scg10, and Chl1) in CST neurons, although this regenerative response did not contribute to sprouting or regeneration of CST axons damaged in the spinal cord at the time of LPS application [48]. It would be interesting to determine if the application of LPS (or another inflammatory agent) into the cervical enlargement would elicit a response in LDPT neurons after thoracic axotomy that mirrors the response observed in TPS neurons. The role of the inflammatory response in axonal regeneration has also been demonstrated for retinal ganglion cell (RGC) axons and the dorsal root process of dorsal root ganglion (DRG) neurons. Both classes of axon grow poorly within peripheral nerve grafts unless the site of axotomy is near their cell bodies of origin [67]. This regenerative response is enhanced by an inflammatory response elicited by lens injury or other perturbation (RGCs), or injection of an inflammatory agent near DRG neurons [59,62,68]. Taken together, these findings, in combination with our previous study [15], support the hypothesis that an inflammatory response may play an important role in the ability of CNS neurons to mount a regenerative response.

If on the other hand, the post-injury response observed in the TPS neurons was primarily the result of the axotomy occurring closer to the cell body of the neuron [2,47,56,57] it is reasonable to expect that SCI causing axotomy nearer to the cell body of LDPT neurons should mount a cellular response similar to TPS neurons after low thoracic SCI. Previous work with SSNs supports this idea. An early regenerative response, marked by the up-regulation of growth associated genes (*i.e. *Atf3, Jun, and Gap43), occurs in CST neurons following axotomy near the cell body that is not found after spinal axotomy [21,57]. An early regenerative response is also seen within rubrospinal tract neurons following cervical axotomy that does not occur after thoracic axotomy [47].

### Phenotypic Differences Between Uninjured LDPT and TPS Neurons

A third possibility to explain the differences in the cellular response post-axotomy of TPS and LDPT neurons is a basic difference in the normal expression of genes involved in apoptosis, neuroprotection, regeneration associated genes, etc. that could influence the cellular response post-axotomy. As discussed above, large differences were found when the uninjured control data from both the LDPT and TPS neurons were compared. These results are both interesting and unexpected, although there are a number of fundamental differences between these two populations of PS neuron. One obvious difference is the length of the axonal projections of these two classes of neuron and the differing metabolic needs to maintain them. For instance, many neurodegenerative diseases result from axonal transport defects, and long tract axons are the population susceptible to these diseases [69]. LDPT neurons are located within the intermediate gray matter of the cervical enlargement, and project their axons caudally for many spinal segments terminating within the intermediate gray matter of the lumbosacral enlargement [10,70]. TPS neurons, on the other hand, arise from the thoracic spinal gray matter, and project their axons for shorter distances in either the rostral or caudal direction [10,70]. These differences in axonal length, alone, could explain the generally higher expression of many of the neurotrophic genes and their receptors that were found in LDPT neurons compared to their TPS counterparts. Of the genes demonstrating a significant difference in expression between the cervical (LDPT neurons) and thoracic levels (TPS neurons) of the spinal cord, over 90% show a significantly higher expression in LDPT neurons. These genes included potent neurotrophic factors (Artn, Ntf5), neurotrophic factor receptors (Cntfr, Gfra1, Gfra2, Lifr, Ntrk1, and Ntrk2), or other molecules known to be involved with neural protection and cellular stress/axonal maintenance (Hspb1, Nf1, Zfp91). Therefore the increased expression of these neurotrophic agents and neurotrophic/growth factor receptors in LDPT neurons may be related to their axonal length, function, and maintenance. A second potential difference between these two subclasses of PS neuron, associated with the length of their projections, is the greater possibility of collateral projections of these axons between the cell body and point of axotomy. The lack of or down-regulation in the expression of the effected genes may be the result of "sustaining collaterals". Such collaterals could interfere with a significant regenerative response, because the neuron is still receiving adequate trophic support. Similarly, the axonal projections of many SSNs, such as the CST, form collateral projections rostral to the spinal cord, and this may be one reason for the lack of regenerative response of Purkinje cell axons, even with axotomy close to the cell body, because of their prominent recurrent collaterals [71].

The significant difference in expression of the reference genes (Rplp1, Ldha, Hprt, and Rpl13a) is also intriguing. These data are unlikely to be a plate loading or cell concentration artifact, since a closer examination of the -ΔΔCp values reveals two of the reference genes, Rplp1 and Rpl13a, to be higher in LDPT than TPS neurons, while the other two reference genes, Ldha and Hprt, are found to be more highly expressed in TPS than in LDPT neurons. If the difference in expression of the reference genes was the result of a plate or cell loading artifact, these differences in expression should be homologous, with all four reference genes having change in the same direction (all significantly increased or decreased). However both ribosomal protein genes, ribosomal protein, large P (Rplp1) and ribosomal protein L13a (Rpl13a) were increased in LDPT neurons when compared to TPS neurons, while lactate dehydrogenase A (Ldha) and hypoxanthine phosphoribosyltransferase 1 (Hprt) demonstrated a higher expression in TPS neurons. Moreover, other PCR plate controls showed similar values for the LDPT and TPS findings (data not shown). We conclude that these differences in our reference genes between samples indicate a further phenotypic difference between these two subsets of PS neurons.

### Therapeutic Implications for LDPT Neurons

The lack of a regenerative response and massive down-regulation of genes that occurs in LDPT neurons after T9 level SCI, is in stark contrast to that found in TPS neurons after the same injury. The lack of any indication of a cell death response either immediately or during the first month post-injury is similar to what is found after spinal axotomy for most SSNs where neuronal atrophy occurs over time, and cell loss occurs slowly, if at all [72-74].

Previous studies have documented the advantage PS neurons have over SSNs, in their regenerative ability after spinal cord injury [3,7,74,75]. In all of these instances, PS neurons were near the lesion site and able to regenerate their axons into permissive environments such as peripheral nerve implants or other trophic molecule enriched implants. Our findings in the current study, while surprising, suggest that following thoracic axotomy, the response of LDPT neurons may be more similar to the response seen after spinal axotomy in SSNs. It is presently unknown if LDPT neurons atrophy or are lost at longer survival times post-thoracic axotomy. From previous findings by others described above, a stronger regenerative response might occur if LDPT neurons were axotomized and/or an inflammatory response occurred nearer their cell bodies at the same time as axotomy

To determine the neurotrophic or growth factor(s) that would be most effective in potentially fostering a regenerative response, we analyzed surface receptor and growth factor expression profiles (Figure 4). We found the simultaneous down-regulation of three out of the four receptors of the GDNF family (Gfra1, Gfra2, Gfra3), as well as the down regulation of Ntrk1 (TrkA) and the Adcyap1r1 receptor. Iannotti and colleagues have shown GDNF to enhance axonal growth of PS neurons within implants, and that intrathecal application of GDNF to a SCI lesion site is neuroprotective [8]. However, in the case of thoracic axotomy of LDPT neurons, our data indicate that GDNF is unlikely to be the neurotrophic agent of choice. Additionally the down-regulation of the NGF receptor, Ntrk1 (TrkA) argues against the use of nerve growth factor (NGF) as a potential therapeutic agent. In fact, supplying a neurotrophic agent to a neuron without the appropriate receptors can be harmful. For instance, when sympathetic neurons, expressing p75 and TrkA surface receptors, were presented with the neurotrophic molecule BDNF, subsequent binding of BDNF to the p75NTR without binding to TrkB ultimately led to the death of the neurons via p75NTR induced apoptosis [76,77].

Even though our present study revealed a down-regulation of three receptors of the GDNF family [78,79] and Ntrk1, genes for other growth factor receptors that did not demonstrate a change in expression may provide clues as to which neurotrophic agents might prove useful for LDPT axonal regeneration. Our analysis revealed no significant difference in the expression of Lifr, a receptor for LIF and a co-receptor for CNTF, or Cntfr, the primary receptor for CNTF. Previous studies have demonstrated both LIF and CNTF to be important growth factors responsible for stimulating axonal regeneration [80-82]. Additionally we found no significant change in the post-axotomy expression of Ntrk2 (TrkB; the primary BDNF receptor) and Ntrk3 (TrkC/NT-3; neurotrophin 3). These neurotrophins have also been reported to be highly neuroprotective and/or promote axonal sprouting/regeneration in other classes of neurons, including SSNs [3,74,75]. Additionally, we found that Lifr, Cntfr, and Ntrk2 are significantly more highly expressed in uninjured LDPT neurons than in TPS neurons. This may indicate that these previously discussed therapeutic agents may be especially beneficial for LDPT neurons. The nominal down regulation of Pycard and Casp2, lack of a significant effect on the expression of Atg9a, Atg9b, Bax, or Tp53 and lack of any TUNEL immunostaining post-SCI, strongly suggests that LDPT neurons do not undergoing apoptosis during the first month post-thoracic SCI. These findings suggest that delivering the suggested neurotrophic agents at a higher level to LDPT neurons than are normally present post-SCI may have potential therapeutic benefit during the first month following thoracic SCI.

## Conclusions

Previous studies have demonstrated the ability of PS axons to grow into peripheral nerve implants and neurotrophin enriched bridges, form functional new neuronal bypass circuits around an incomplete lesion, and cross the midline to form new circuits [7,8,83-85]. Additionally, our previous findings show an initial robust intrinsic response of TPS neurons to a low T9 level axotomy [15]. The current study, combining qRT-PCR with immunofluoresence data demonstrate that LDPT neurons respond more like SSNs than TPS neurons following a low thoracic axotomy. The down-regulation of many genes, including growth factors, neurotrophic/growth factor surface receptors, and cell death elements, is surprising based on the multitude of reports indicating the robust regenerative response of PS neurons. However, this intrinsic cellular response of LDPT neurons may result from the axotomy occurring many segments from LDPT neurons. The current study also demonstrates a fundamental difference in the baseline expression of many of the genes evaluated when LDPT and TPS neurons are compared. Our data indicate that LDPT neurons will need to be treated in a manner completely different than their thoracic counterparts following spinal injury, since there are significant differences in their post-axotomy response to thoracic SCI (see Figure 4).

## Authors' contributions

DJS and FAM designed the study; JRS performed all animal FG surgeries and qRT-PCR experiments. JRS, FAM, and DJS all participated in data analysis and interpretation. JRS wrote the initial manuscript. JRS, FAM, and DJS all reviewed edited and approved the final version of this manuscript.

## Supplementary Material

Additional file 1 **Complete list of all the Genes examined by PCR**. List containing the names and gene symbols for all the genes that were screened for using PCR. Click here for file
