# Supplementary material for: Long descending cervical propriospinal neurons differ from thoracic propriospinal neurons in response to low thoracic spinal injury
Source: BMC Neurosci. 2010 Nov 23;11:148. doi: 10.1186/1471-2202-11-148 (PMC3001741; doi:10.1186/1471-2202-11-148)
Supplement: Additional file 1 — Complete list of all the Genes examined by PCR. List containing the names and gene symbols for all the genes that were screened for using PCR. [file 1471-2202-11-148-S1.DOC]

| **Symbol** | **Title** |
| --- | --- |
| Abcb5 | ATP-binding cassette, sub-family B |
| Actb | Actin, beta |
| Adcyap1r1 | Adenylate cyclase activating polypeptide 1 receptor 1 |
| Akt3 | v-akt murine thymoma viral oncogene homolog 3 |
| Arg1 | Arginase 1 |
| Artn | Artemin |
| Atf3 | activating transcription factor 3 |
| Atg9a | ATG9 autophagy related 9 homolog A |
| Atg9b | ATG9 autophagy related 9 homolog B |
| Atrn | Attractin |
| Bax | Bcl2-associated X protein |
| Bcl2 | B-cell leukemia/lymphoma 2 |
| Bdnf | Brain derived neurotrophic factor |
| Casp2 | Caspase-2 |
| Cbln1 | Similar to cerebellin 1 precursor protein |
| Cckar | Cholecystokinin A receptor |
| Cntf | Ciliary neurotrophic factor |
| Cntfr | Ciliary neurotrophic factor receptor |
| Crem | cAMP responsive element modulator |
| Crh | Corticotropin releasing hormone |
| Crhbp | Corticotropin releasing hormone binding protein |
| Crhr1 | Corticotropin releasing hormone receptor 1 |
| Crhr2 | Corticotropin releasing hormone receptor 2 |
| Cx3cr1 | Chemokine (C-X3-C) receptor 1 |
| Cxcr4 | Chemokine (C-X-C motif) receptor 4 |
| Dbh | dopamine beta-hydroxylase |
| Fas | Fas (TNF receptor superfamily, member 6) |
| Fgf2 | Fibroblast growth factor 2 |
| Fgf9 | Fibroblast growth factor 9 |
| Fgfr1 | Fibroblast growth factor receptor 1 |
| Flna | filamin A |
| Fos | FBJ murine osteosarcoma viral oncogene homolog |
| Frs2 | Fibroblast growth factor receptor substrate 2 (predicted) |
| Frs3 | Fibroblast growth factor receptor substrate 3 |
| Fuk | fucokinase |
| Fus | Fusion, derived from t(12;16) malignant liposarcoma (human) |
| Galr1 | Galanin receptor 1 |
| Galr2 | Galanin receptor 2 |
| Gdnf | Glial cell line derived neurotrophic factor |
| Gfra1 | Glial cell line derived neurotrophic factor family receptor alpha 1 |
| Gfra2 | Glial cell line derived neurotrophic factor family receptor alpha 2 |
| Gfra3 | Glial cell line derived neurotrophic factor family receptor alpha 3 |
| Gmfb | Glia maturation factor, beta |
| Gmfg | Glia maturation factor, gamma |
| Grm4 | glutamate receptor, metabotropic 4 |
| Grpr | Gastrin releasing peptide receptor |
| HcRt | Hypocretin |
| Hcrtr1 | Hypocretin (orexin) receptor 1 |
| Hcrtr2 | Hypocretin (orexin) receptor 2 |
| Hspb1 | Heat shock 27kDa protein 1 |
| Il10 | Interleukin 10 |
| Il10ra | Interleukin 10 receptor, alpha |
| Il1b | Interleukin 1 beta |
| Il1r1 | Interleukin 1 receptor, type I |
| Il6 | Interleukin 6 |
| Il6ra | Interleukin 6 receptor, alpha |
| Il6st | Interleukin 6 signal transducer |
| Itb1 | Integrin Beta 1 |
| Itga6 | integrin, alpha 6 |
| Lif | Leukemia inhibitory factor |
| Lifr | Leukemia inhibitory factor receptor |
| LOC685671 | Myocyte enhancer factor 2a |
| Maged1 | Melanoma antigen, family D, 1 |
| Maob | monoamine oxidase B |
| Mc2r | Melanocortin 2 receptor |
| Mt3 | Metallothionein 3 |
| Myc | Myelocytomatosis viral oncogene homolog (avian) |
| Nell1 | NEL-like 1 (chicken) |
| Nf1 | Neurofibromatosis 1 |
| Ngfb | Nerve growth factor, beta |
| Ngfr | Nerve growth factor receptor (TNFR superfamily, member 16) |
| Ngfrap1 | Nerve growth factor receptor (TNFRSF16) associated protein 1 |
| Notch3 | Notch homolog 3 |
| Npffr2 | Neuropeptide FF receptor 2 |
| Npy | Neuropeptide Y |
| Npy1r | Neuropeptide Y receptor Y1 |
| Npy2r | Neuropeptide Y receptor Y2 |
| Nr1i2 | Nuclear receptor subfamily 1, group I, member 2 |
| Nrap | nebulin-related anchoring protein |
| Nrg1 | Neuregulin 1 |
| Nrg2 | Neuregulin 2 |
| Ntf3 | Neurotrophin 3 |
| Ntf5 | Neurotrophin 5 |
| Ntrk1 | Neurotrophic tyrosine kinase, receptor, type 1 |
| Ntrk2 | Neurotrophic tyrosine kinase, receptor, type 2 |
| Oaz1 | ornithine decarboxylase antizyme 1 |
| Pdgfra | Platelet Derived Growth Factor AA |
| Ppyr1 | Pancreatic polypeptide receptor 1 |
| Pspn | Persephin |
| Ptger2 | Prostaglandin E receptor 2, subtype EP2 |
| Pycard | PYD and CARD domain containing |
| Rhoq | ras homolog gene family, member Q |
| Sat1 | spermidine/spermine N1-acetyltransferase 1 |
| Slc27a3 | solute carrier family 27, member 3 |
| Slc6a11 | solute carrier family 6, member 11 |
| Stat1 | Signal transducer and activator of transcription 1 |
| Stat2 | Signal transducer and activator of transcription 2 |
| Stat3 | Signal transducer and activator of transcription 3 |
| Stat4 | Signal transducer and activator of transcription 4 |
| Tacr1 | Tachykinin receptor 1 |
| Tfg | Trk-fused gene |
| Tgfa | Transforming growth factor alpha |
| Tgfb1 | Transforming growth factor, beta 1 |
| Tgfb1i1 | Transforming growth factor beta 1 induced transcript 1 |
| Tgfbr1 | transforming growth factor, beta receptor 1 |
| Tnfrsf5 | Tumor necrosis factor receptor superfamily, member 5 |
| Tp53 | Tumor protein p53 |
| Ucn | Urocortin |
| Vgf | VGF nerve growth factor inducible |
| Xiap | X-linked inhibitor of apoptosis |
| Zfp110 | Zinc finger protein 110 |
| Zfp53 | Zinc Finger Protein 53 |
| Zfp91 | Zinc finger protein 91 |
